# Cardiovascular Endurance, Body Mass Index, Physical Activity, Screen Time, and Carotenoid Intake of Children: NHANES National Youth Fitness Survey 

**DOI:** 10.1155/2016/4897092

**Published:** 2016-09-27

**Authors:** Joan A. Vaccaro, Fatma G. Huffman

**Affiliations:** Department of Dietetics and Nutrition, Florida International University, 11200 SW 8th Street, MMC AHC5 306, Miami, FL 33199, USA

## Abstract

*Background*. Approximately 17% of children aged 6–11 years were classified as obese in the United States. Obesity adversely affects physical functioning and leads to reduced quality of life. Heart function for overweight and obese children has not been reported.* Methods*. Data for this study were from NHANES National Youth Fitness Survey (NNYFS) conducted in conjunction with the National Health and Nutrition Examination Survey (NHANES) in 2012. This study used data from children aged 6–12 (*N* = 732) that had the cardiorespiratory endurance measure, body mass index for age and sex, and dietary data (*N* = 682). Cardiovascular endurance was estimated by heart rate reserve.* Results*. Compared to the highest percentile of heart rate reserve, those in the first percentile had 3.52 (2.36, 5.24) odds and those in the second percentile had 3.61 (1.84, 7.06) odds of being in the overweight/obese as compared to the under/normal weight category. Considering the highest percentile, boys had a heart rate reserve of 35%, whereas girls had a heart rate reserve of 13% (less than half that of boys).* Conclusion*. Having an overweight or obese classification for children in this study demonstrated a compromise in cardiovascular endurance. Parental awareness should be raised as to the detrimental consequence of overweight and heart health.

## 1. Introduction

Approximately 17% of children aged 6–11 were classified as obese in the United States [1]. Children who are classified as obese are at greater risk for cardiovascular disease in later life than their counterparts [2]. Obesity adversely affects physical functioning and leads to reduced quality of life [3]. Prevention of excess weight-gain should be addressed with population interventions that focus on achieving a healthy diet, recommended physical activity, and energy balance [3]. Among the key lifestyle behaviors, low fruit and vegetable intake, little physical activity, and high screen time have all been attributed to childhood obesity worldwide [4]. Regardless of sociodemographic group, no children from a US representative sample of children age 2–18 years met the vegetable recommendations and only children aged 2–5 years met the recommended intake of fruit [5]. Less than half of children aged 3–17 years are meeting the United States Department of Health and Human Services' physical activity guidelines of 60 minutes or more per day of moderate to vigorous physical activity [6]. Although the majority of US sample of children aged 9–13 years reported some form of physical activity in the past 7 days, non-Hispanic Black and Mexican-American children had lower participation in free-play and organized sports [7]. It has been suggested that participation in organized sports may be lower in disadvantaged groups who live in unsafe neighborhoods and/or who have difficulty with transportation [7, 8]. Children from economically disadvantaged family have fewer physical activity opportunities than their peers from economically stable families [8]. Stronger government policies are recommended to increase the frequency, intensity, and duration of physical activity and physical education at public schools for elementary and secondary education [9]. Approximately 70% of US elementary schools require physical education and among them less than half evaluate the success of their program by student fitness scores [10]. Screen time averages 7.5 hours per day based on a survey of 8–18-year-old children [11].

The detrimental effects of childhood obesity in adulthood have been reported; however, the immediate effect of overweight/obesity on heart function has had little attention. Heart rate reserve (HRR), the difference between maximum and resting heart rate, is an indicator of physical functioning. Based on the adverse outcomes of obesity, the following hypothesis was tested: The overweight/obese group will have higher odds of low HRR, high screen time, no physical activity (in the past 7 days), and low carotenoid intake per calorie (a measure of green and orange vegetables), adjusting for race/ethnicity, sex, and age.

## 2. Methods

### 2.1. Data Collection

The National Health and Nutrition Examination Surveys (NHANES), under the auspices of the Center for Disease Control and Prevention (CDC) acquired health-related factors from representative samples of noninstitutionalized persons of all ages in two-year cycles. NHANES National Youth Fitness Survey (NNYFS) was conducted in conjunction with the National Health and Nutrition Examination Survey (NHANES) in 2012 in response to the need for data on physical activity and fitness levels in children and adolescents. NNYFS uses the same primary sampling units and procedure as NHANES for a noninstitutionalized, resident population of children aged 3–15 years in the United States within the 50 states and the District of Columbia. The first stage of sampling was from the counties where 15 Primary Sampling Units (PSU) were selected. The second stage selection was from census blocks and included 24 area segments. Next, a screening procedure was performed to establish dwelling units that were suitable for screening (excluding institutions such as Job Corp centers, workers' group living facilities, residential treatment, adult group-homes, and college housing). The screener's primary objective was to determine whether any children in the household were eligible for the interview and examination. After matching the preset age-sex probability, NNYFS included three levels of data collection: (1) a household screening interview (or screener); (2) an in-home personal interview where data were collected for demographics; and (3) health, nutrition, and physical activity and physical examinations. These physical measurements and fitness tests were conducted at the mobile examination centers (MEC). The fitness tests included standardized measurements of core upper and lower body muscle strength, as well as a measurement of cardiovascular fitness by walking and running on a treadmill [12]. Sample weights were designed to compensate for differences in the probability of selection, reduce nonrespondent bias, and better match the US census population's sociodemographics [12].

### 2.2. Participants

Data used in this study were publically available. There were 1640 children and adolescents interviewed, 1576 aged 3–15 years examined. Cardiorespiratory endurance was evaluated for children 6–11 years of age. This study used data from children aged 6–12 (*N* = 732) that had the cardiorespiratory endurance measure (*N* = 682). Note: age at screening was 6–11 years at the time of testing some children reached 12 years. The race/ethnicity category “Other” was excluded, due to insufficient number in this category. The final sample size was 614 children who completed the cardiovascular endurance test and of the following self-reported race/ethnicity categories: 103 Mexican Americans (MA), 103 Other Hispanics (OH), 156 non-Hispanic Blacks (NHB), and 252 non-Hispanic Whites (NHW). The NNYFS protocol was approved by the NCHS Research Ethics Review Board (ERB) and the rights and welfare of the NNYFS participants were protected by the ERB [13]. The informed consent process for NNYFS followed the procedures established for NHANES [13]. Documented signed consent was obtained from each sample person's parent or guardian prior to the household interview and the examination [13]. Children aged 7–15 provided additional signed assents to participate in the examination [13].

### 2.3. Procedure

#### 2.3.1. General Interview

The person and household interview was conducted to determine sociodemographic factors and health history. Upon completion of the general interview, the participant was instructed to report to the MEC for the dietary interview and examination components.

#### 2.3.2. Dietary Interview

The examination protocol and data collection methods are fully documented in the NNYFS dietary interviewer procedures manual (http://www.cdc.gov/nchs/data/nnyfs/Dietary_Interview.pdf).

Interviews of children aged 6–8 years were conducted with a proxy and with the child present to assist in reporting intake information. Children aged 9–12 were asked to provide their own data, with the assistance of an adult household member familiar with the child's intake. Dietary interviewers conducted interviews in English and Spanish. Translators were used to conduct interviews in other languages. The in-person interview was conducted in a private room in the NNYFS' MEC. A set of measuring guides (various glasses, bowls, mugs, drink boxes and bottles, household spoons, measuring cups and spoons, a ruler, thickness sticks, bean bags, and circles) was available in the MEC dietary interview room for the participant to estimate portion size (NNYFS Measuring Guides for the Dietary Recall Interview).

Dietary interview data for the NNYFS were collected in the MEC using USDA's dietary data collection instrument, the Automated Multiple-Pass Method (AMPM) (http://www.ars.usda.gov/ba/bhnrc/fsrg) following the same procedure as NHANES first-day 24-hour recall, an interviewer-administered, computerized recall method that uses a 5-step interview process. The NNYFS collected one, detailed 24-hour recall, but did not include a telephone interview as used by NHANES.

#### 2.3.3. Anthropometric Measures and Overweight/Obesity Variable

All body measures were taken in a private examination room with light clothing and no shoes. Height and weight were used to calculate age-sex body mass index (zBMI) and used to form the categories based on the CDC Growth Charts (underweight: <5%, normal weight: 5%–<85%, overweight: 85%–<95%, obese ≥95%) [14].

#### 2.3.4. Cardiovascular Endurance

This test is administered to children who passed a “nurse review” where parent/guardian were asked if their child had any physical disabilities, surgeries, or other circumstances that would exclude them from participation. Details of exclusion criteria can be found online under the MEC operations, safety precautions at http://www.cdc.gov/nchs/data/series/sr_02/sr02_163.pdf. The cardiovascular endurance component was conducted in the MEC by a team trained professionals: nurse practitioners and exercise physiologists, half of whom were bilingual. Staff had CRP certification and they were trained to follow a standard protocol. Biological data were entered automatically into an* Integrated Survey Information System*. This computer-based infrastructure supported data collection, data editing, quality control, and the delivery of NYFS data. The equipment was calibrated and participants wore standard athletic shoes (provided). These standardized procedures allowed maximum precision of endurance performance measures of completers. It should be noted that endurance performance, measured in young children, is an estimation and not an accurate account of aerobic capacity. The speeds and inclines used in the test were based on aerobic capacity of children by age and sex. Rather than using maximum heartrate as an indicator of aerobic capacity, the endurance performance test allows the child to reach their maximum state of physical exhaustion. Detailed information about assessment of cardiovascular endurance is found at http://www.cdc.gov/nchs/data/nnyfs/treadmill.pdf. In summary, protocols were specific to age ranges. There were three separate protocols: ages 6-7, 8-9, and 10–12. Each protocol included a 5–12-minute test which was conducted in 7 stages: a warm-up phase; testing phases with a maximum incline of 12.5%; and a cooldown phase. Protocols were designed so increases in speed and incline corresponded to the child's fitness/endurance level from a walking to jogging pace. The test was stopped immediately if the participant complained of pain or discomfort. The test was also stopped for participants who after repeated coaching did not follow directions. Heart rate was measured by electrodes. Those who stopped the test early due to discomfort were not considered completers and were not included in the analysis for this study.

### 2.4. Physical Fitness, Physical Activity, Sedentary Behavior, and Dietary Variables

Cardiovascular endurance was estimated by HRR. The difference between the heart rate at the end of test and resting heart rate was calculated as HRR. The measure was not linear and could not be transformed to approach normality; therefore quartiles of HRR were used ranging from <32, 32–38.9, 39–48, and >48 bpm. Physical activity was assessed by an affirmative answer to “have you participated in any physical activity in the past 7 days?” where physical activity was explained. Screen time as assessed by participants' responses to the total number of hours engaged in viewing television, playing video games, or using the computer per day. Categories were collapsed to <1 hour, 1 hour, 2 hours, ≥3 hours per day. The total number of hours of sedentary behavior per day was not measured for this study. Screen time and physical activity were considered for after-school hours, only.

In order to assess the determinants of overweight and obesity, categories were collapsed to under/normal weight and overweight/obese. The variable for carotenoids was calculated by combining alpha carotene, beta carotene, lycopene, and lutein/zeaxanthin. The variable for high carotenoids/low carotenoids was formed by the 75th percentile of total carotenoids/Kcal.

### 2.5. Statistical Analysis

A sample analysis plan was formed using the strata and units and applying the sample weight from the mobile examination center (MEC) using the Statistical Package for the Social Sciences, IBM SPSS with the complex sample analysis module [15]. Continuous variables were tested for normality using the Kolmogorov-Smirnov test. Variables not achieving linearity, even with transformation, were converted to categorical variables. Descriptive statistics were run for race/ethnicity using the Chi-squared test and the 95th percent confidence intervals of the standard error to determine differences among subgroups. Logistic regression analysis for complex samples was used to test the main hypothesis with overweight/obesity as the outcome. A *p* value of < 0.005 was considered significant.

## 3. Results

The overweight/obesity characteristics were compared by race/ethnicity (Table 1) and by sex (Table 2). There was a trend for a higher percent of NHW reporting participation in physical activity in the past 7 days (90% as opposed to approximately 84% for other racial/ethnic groups *p* = 0.075). Compared to other groups, BNH and OH had a higher percent of screen time in the ≥3 hours/day category as compared to MA and NHW; albeit, the relationship was not significant at the *p* < 0.05 level. Other measures of overweight/obesity characteristics were not significantly different by race/ethnicity.

Overweight/obesity characteristics by sex are shown in Table 2. Girls had a significantly higher percent in the 75th percentile of carotenoids/Kcal as compared to boys (28% versus 22%).

Considering the highest percentile, boys had a HRR of 35%, whereas girls had a HRR of 13% (less than half that of boys). There were no differences in zBMI category, participation in PA or school sports by sex, and screen time by sex.

The final model for the determinants of overweight/obesity is shown in Table 3. The model explains 14.5% of overweight/obesity as determined by the Nagelkerke pseudo *R*-squared with a significance of *p* = 0.05. The hypothesis was partially supported, in that HRR and screen time were determinants of obesity. Carotenoids could not be fit in the final model and physical activity was not a significant determinant (*p* = 0.391). Race/ethnicity (*p* = 0.378), sex (*p* = 0.064), and age (*p* = 0.146) were not significantly related to overweight/obesity. Compared to the highest percentile of HRR, those in the first percentile had 3.52 (2.36, 5.24) odds and those in the second percentile had 3.61 (1.84, 7.06) odds of being in the overweight/obese as compared to the under/normal weight category. There was no significant difference between the third and fourth percentile [OR = 1.86  (0.77,4.47)]. Compared to the highest level of screen time (≥3 hours/day), those in the first percentile (<1 hour/day) had 0.51 (0.30, 0.88) odds and those in the second percentile (1 hour/day) had 0.38 (0.18, 0.84) odds of being in the overweight/obese category as compared to the under/normal weight category. Screen time of 2 hours/day was not significantly different from ≥3 hours/day [OR = 0.92  (0.47,1.78)].

Chi-squared test for complex samples was preformed to assess the relationship between level of zBMI category and HRR. Participants in the normal zBMI category had the greatest percent in the highest HRR (best functioning) [30.6 (24.7–37.2)] compared to those in the overweight category [18.0 (13.4, 23.7)] and those in the obese category [9.4 (5.8, 15.1)] (overall significance *p* = 0.001, data not shown). Before combining underweight with normal weight, we ran statistics (weighted) and found no significant difference between underweight and normal weight with respect to outcome variables. We surmised that the unweighted count for underweight may be too small to be representative of underweight children in the US.

## 4. Discussion

This study compared a measure of physiological function, HRR for a representative sample of US children aged 6–12 year with obesity indicators and risk factors. The major finding was that children in the overweight and obese categories were more-likely to have HRR in the lower quartiles as compared to children in the underweight/normal weight category. The findings indicate that being overweight may reduce the physiological function of the heart. There have been no publications comparing HRR with body mass for age and sex in children. Prior research focused on pulmonary function differences between obese and normal weight adolescents and adult. The advantage of exercise for children is the concomitant increase in HRR along with increased energy expenditure which reduces the risk for obesity. Conversely, it has been shown that expiratory volume and, to a lesser extent, total lung capacity are reduced in individuals with obesity [16]. Fat distribution is a confounder for obesity since fat distribution around the abdomen may create airway resistance [16]. Fat distribution was found to alter cardiorespiratory function for obese compared to normal weight adolescents [17].

Obesity and obesity determinants were assessed by race/ethnicity and by sex. There were no differences in obesity or obesity indicators by race/ethnicity for this study. One explanation is that completers of a cardiovascular endurance test differ from the general population. Another plausible explanation is that the relationship of race/ethnicity and obesity in children is not clear and is confounded by socioeconomic, cultural, and biological factors [18]. Considering sex, there were no differences zBMI category, participation in PA or school sports, and screen time. Sex was associated with HRR, where boys had a higher percent in this highest quartile of HRR. This study found no other indicators of obesity was related to HRR for girls and boys combined, although screen time was correlated with BMI for age and sex (zBMI). Higher percent of overweight and obese children reported engaging in 3 or more hours of screen time (a combination of watching television, playing video games, and using the computer). There were no significant differences in zBMI for 2 hours versus 3 or more hours of screen time per day. These findings are corroborated by the American Academy of Pediatrics' recommendation of no more than 2 hours of screen time per day [19, 20]. Screen time was correlated to BMI for age and sex for boys but not girls aged 7–12 [21]. Laurson et al. [21] found habitual physical activity was positively correlated with age and sex BMI for children aged 7–12 for girls but not for boys which is in contrast to our study. This could be due to the nature of the variable for physical activity. For the NNYFS used in the current study, physical activity was self-reported for any within the past 7 days, whereas Laurson and colleague's study [21] assessed physical activity by pedometer over the past 7 days and meeting recommendations or not by steps (13,000/day for boys and 11,000 per day for girls).

Another obesity indicator is the consumption of fruits and vegetables. The current study did not find an association with carotenoid intake, adjusted by energy (carotenoid/Kcal), a pseudo-measure of fruit and vegetable intake, with BMI for age and sex. Our results were in agreement with a systematic review, whereby fruit and vegetable intake was associated with body weight loss for adults but not for children in a review of longitudinal studies [22]. The author concluded that factors such as total calorie intake, sedentary behavior, and physical activity may have a greater influence on weight loss for children, that is, increasing fruits and vegetable without decreasing total calories [22]. Whether children willingly substitute fruit and vegetables for high-calorie snacks and meal options may depend on their direct experience (such as participation in school taste-tests and school/community gardens) [23]. This information was not available and may have confounded the association of carotenoid/Kcal and body mass index for age and sex. There were significantly more girls that consumed the highest percent of carotenoids/Kcal for the current study. Our results were in agreement with a systematic review, whereby Rasmussen and colleagues [24] reported sex-differences in fruit and vegetable consumption with girls having a higher intake than boys.

Some limitations of the current study should be considered. HRR was measured by the differences between heart rate after performing the cardiovascular endurance test and resting heart rate. The measure assumes the maximum heart rate was achieved at the end of the test and this may not be true for all participants. The difference between completers and noncompleters was not considered; however, this study compared only those children who were asked to stop at the end of the test (completers) and not those who had to terminate the test early. Screen-time was considered sedentary activity and there are active computer games. For example, Mellecker and McManus [25] found an increase in heart rate for children involved in active gaming as compared to those with sedentary computer games. There were no data collected on other forms of sedentary behavior, such as playing board games, reading, or doing homework. The number of hours in school is standard for children aged 6–12 years who attend public school. This could differ when considering those children home-schooled or attending a public school. The total number of hours of sedentary behavior per day was not measured for this study. Screen time and physical activity were considered for after-school hours, only.

Total carotenoid intake (not considering supplements) was used to estimate fruit and vegetable intake rather than a direct measure. The strength of using carotenoid intake was adjusting it per calorie. A major strength of this study was the use of a national representative sample of US children with cardiovascular endurance data measured for the first time by this new survey and testing the association of physiological functioning with obesity risk factors and indicators.

## 5. Conclusion

Screen time was positively associated with zBMI for both boys and girls. Having an overweight or obesity classification for children in this study demonstrates a compromise in cardiovascular endurance, as measured by HRR. Continued efforts in lifestyle education for families are needed to prevent detrimental health consequences for children. Parental awareness should be raised concerning how not just obesity but overweight can be an impediment to physiological functioning of children.

## Figures and Tables

**Table 1:** Overweight/obesity characteristics by race/ethnicity in percent (95th CI).

Variable	MA	OH	NHB	NHW	*p*
Overweight/obese	43.1 (34.7, 51.9)	39.1 (26.3, 53.6)	37.4 (29.0, 46.7)	32.6 (25.0, 41.3)	0.278
High carotenoids/Kcal (75th percentile)	24.0 (18.6, 30.5)	24.6 (16.3, 35.4)	24.0 (18.4, 30.6)	25.1 (19.4, 31.7)	0.970
Participate in school sports (yes)	42.6 (27.5, 59.2)	49.1 (40.3, 57.9)	33.9 (25.4, 43.5)	46.9 (40.3, 53.6)	0.131
Physical activity in past 7 days (yes)	83.0 (72.4, 90.1)	83.2 76.4, 88.4)	83.8 (76.5, 89.1)	89.6 (83.8, 93.5)	0.075
Heart rate reserve (HRR) bpm > 48 bpm	25.8 (17.4, 36.5)	20.1 (14.6, 26.9)	31.7 (24.0, 40.5)	23.0 (16.9, 30.6)	0.207
Screen time/day (total video, computer, television) ≥ 3 hrs/day	16.7 (13.8, 20.0)	28.1 (16.7, 43.4)	33.1 (21.9, 46.7)	16.3 (11.5, 22.4)	0.056

MA: Mexican Americans, OH: Other Hispanics, NHB: non-Hispanic Blacks, and NHW: non-Hispanic Whites.

**Table 2:** Overweight/obesity characteristics by sex in percent (95th CI).

Variable	Male	Female	*p*
Overweight/obese	36.3 (28.6, 44.9)	34.7 (29.3, 40.5)	0.676
High carotenoids/Kcal (75th percentile)	21.7 (17.6, 26.4)	27.9 (22.4, 34.2)	0.036
Participate in school sports (yes)	47.8 (40.1, 55.5)	41.2 (33.9, 49.0)	0.120
Physical Activity in past 7 days (yes)	89.1 (81.5, 93.8)	84.9 (79.5, 89.0)	0.280
Heart rate reserve (HRR) bpm > 48 bpm	35.0 (27.6, 43.2)	13.1 (9.3, 18.3)	<0.001
Screen time/day (total video, computer, television) ≥ 3 hrs/day	19.6 (12.6, 29.2)	20.7 (16.1, 26.1)	0.784

**Table 3:** Odds ratio overweight/obesity versus under/normal weight by age and sex BMI.

Variable	OR (95% CI)	*p*
Heart rate reserve (HRR) bpm	—	<0.001
1st percentile < 32	3.52 (2.36, 5.24)	<0.001
2nd percentile 32–38.9	3.61 (1.84, 7.06)	0.001
3rd percentile 39–48	1.86 (0.77, 4.47)	0.153
4th percentile > 48 (reference)	1.00	—
Screen time (video, computer, television) hrs/day		0.024
<1 hour/day	0.51 (0.30, 0.88)	0.018
1 hour/day	0.38 (0.18, 0 .84)	0.020
2 hours/day	0.92 (0.47, 178)	0.783
≥3 hours/day (reference)	1.00	—
Physical activity (yes) past 7 days	0.77 (0.41, 1.48)	0.391

Adjusted for race/ethnicity, *p* = 0.378, sex, *p* = 0.064, and age, *p* = 0.146.
